# BindingSiteDTI: differential-scale binding site modelling for drug–target interaction prediction

**DOI:** 10.1093/bioinformatics/btae308

**Published:** 2024-05-10

**Authors:** Feng Pan, Chong Yin, Si-Qi Liu, Tao Huang, Zhaoxiang Bian, Pong Chi Yuen

**Affiliations:** Department of Computer Science, Hong Kong Baptist University, Kowloon, 999077, Hong Kong; Department of Computer Science, Hong Kong Baptist University, Kowloon, 999077, Hong Kong; Department of Computer Science, Hong Kong Baptist University, Kowloon, 999077, Hong Kong; Shenzhen Research Institute of Big Data, The Chinese University of Hong Kong (Shenzhen), 518172, China; School of Chinese Medicine, Hong Kong Baptist University, Kowloon, 999077, Hong Kong; School of Chinese Medicine, Hong Kong Baptist University, Kowloon, 999077, Hong Kong; Department of Computer Science, Hong Kong Baptist University, Kowloon, 999077, Hong Kong

## Abstract

**Motivation:**

Enhanced by contemporary computational advances, the prediction of drug–target interactions (DTIs) has become crucial in developing *de novo* and effective drugs. Existing deep learning approaches to DTI prediction are frequently beleaguered by a tendency to overfit specific molecular representations, which significantly impedes their predictive reliability and utility in novel drug discovery contexts. Furthermore, existing DTI networks often disregard the molecular size variance between macro molecules (targets) and micro molecules (drugs) by treating them at an equivalent scale that undermines the accurate elucidation of their interaction.

**Results:**

We propose a novel DTI network with a differential-scale scheme to model the binding site for enhancing DTI prediction, which is named as BindingSiteDTI. It explicitly extracts multiscale substructures from targets with different scales of molecular size and fixed-scale substructures from drugs, facilitating the identification of structurally similar substructural tokens, and models the concealed relationships at the substructural level to construct interaction feature. Experiments conducted on popular benchmarks, including DUD-E, human, and BindingDB, shown that BindingSiteDTI contains significant improvements compared with recent DTI prediction methods.

**Availability and implementation:**

The source code of BindingSiteDTI can be accessed at https://github.com/MagicPF/BindingSiteDTI.

## 1 Introduction

Accurate prediction of drug–target interaction (DTI) constitutes a fundamental process in drug discovery, aiding in the identification of candidate compounds with therapeutic potential (Luo *et al.* 2017, Öztürk *et al.* 2018, Bagherian *et al.* 2021). *De novo* drug development typically spans several years and consumes substantial resources, with extensive reliance on *in vitro* experiments (DiMasi *et al.* 2016, Paul *et al.* 2010).

Predicting DTI remains challenges, particularly for molecules without prior interaction data (Gao *et al.* 2018, Dalkıran *et al.* 2023). The efficacy of predictions hinges on the generalizability of DTI networks and their ability to learn the mechanisms of interaction (Chen *et al.* 2020, Huang *et al.* 2021). Consequently, the development of accurate and generalizable DTI prediction methods continues to be a focus of research.

Previous DTI deep learning approaches can be categorized into representation learning-based methods (Gao *et al.* 2018, Öztürk *et al.* 2019, Abbasi *et al.* 2020, Zheng *et al.* 2020, Nguyen *et al.* 2021, Khojasteh *et al.* 2023, Xia *et al.* 2023) and interaction modelling based methods (Chen *et al.* 2020, Huang *et al.* 2021, Ye *et al.* 2021, Amiri Souri *et al.* 2022, Wang *et al.* 2022, Wu *et al.* 2022, Yazdani-Jahromi *et al.* 2022, Bai *et al.* 2023, Mazzone *et al.* 2023, Nguyen *et al.* 2023).

Representation learning based methods aim to improve the DTI prediction performance by extracting a rich feature of drugs and targets. Recent trends include combining different representations (El-Behery *et al.* 2022, Khojasteh *et al.* 2023) or modelling the feature with multimodalities (Xia *et al.* 2023) to further boost the performance. However, several studies have demonstrated that representation learning-based models often attain high prediction accuracy by overfitting specific representational features (Bai *et al.* 2023). Another category of DTI networks is interaction modelling based methods, these methods mainly depend on forming an interaction map or constructing a knowledge graph to learn the interaction. A recent trend of this methodology is to adopt attention modules to model the hidden relation between drugs and targets (Chen *et al.* 2020, Yazdani-Jahromi *et al.* 2022, Bai *et al.* 2023, Nguyen *et al.* 2023). Current interaction modelling based methods still remain rooms for improvement. For instance, directly constructing a feature map overlooks important domain knowledge, such as chemical properties or structural similarity. Additionally, using a knowledge graph to learn interactions is dependent on the source domain of the training data and may face challenges to generalize to unseen domains. Overall, existing methods demonstrate high accuracy but still contain rooms to generalize to unseen drugs/targets’ interaction.

The substructure of the target interacts with a drug is defined as the binding site (Insel 1988). Binding sites can undergo functional modifications in various scenarios (Kumar and Lukman 2018). Explicitly identifying those binding sites is crucial for DTI prediction (Konc and Janežič 2022, Yazdani-Jahromi *et al.* 2022, Bai *et al.* 2023).

**Figure 1.**
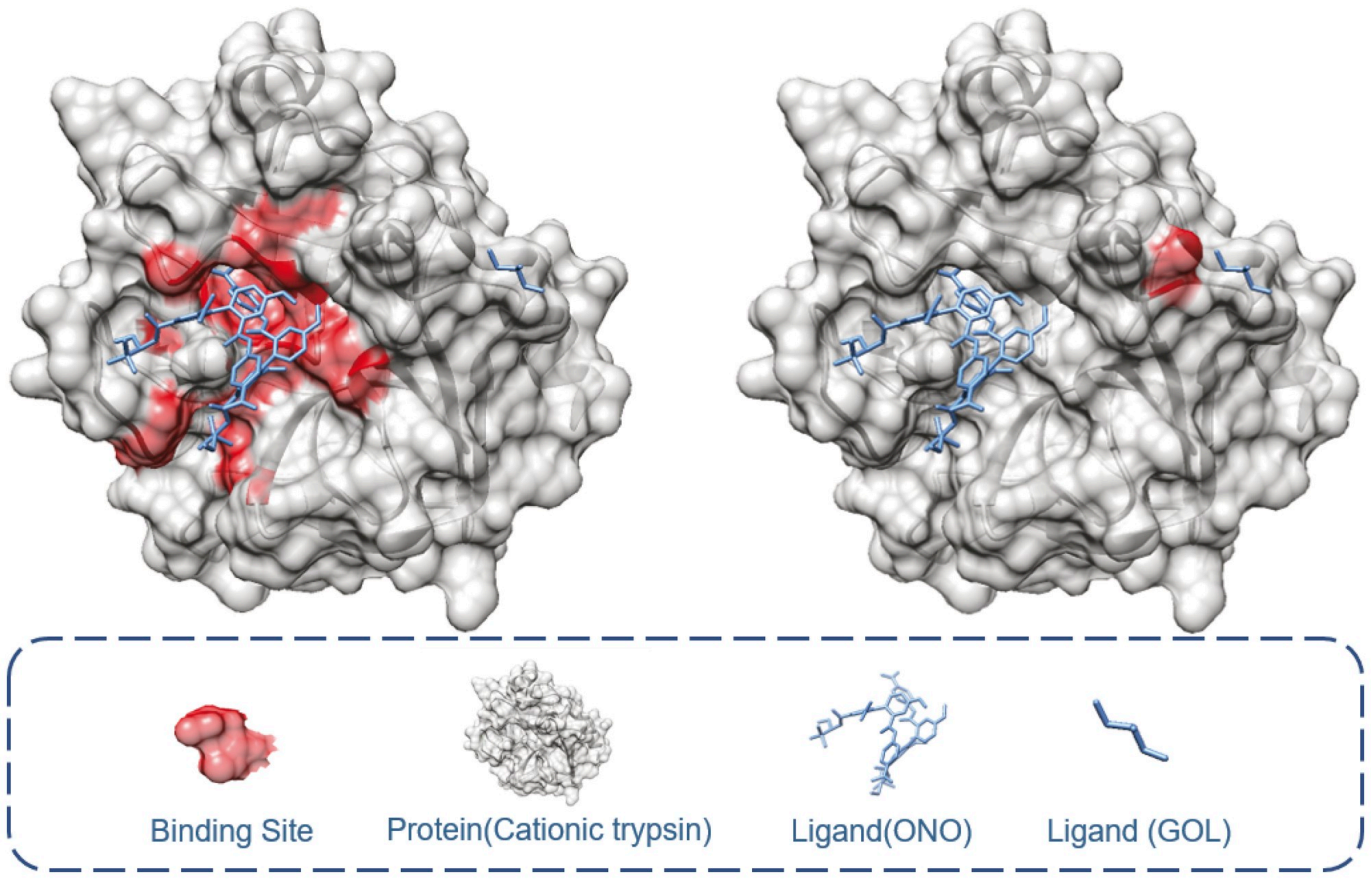
A protein interacts with different drugs, binding sites are extremely different due to the size of drugs.

The sizes of drug and target molecules are important factors that directly influence the occurrence of interactions (Gupta *et al.* 2020). Figure 1 is an example showing that a single protein can interact with different drug molecules of vastly differential molecular sizes of binding sites. Furthermore, several studies have shown that drugs and proteins can bind to multiple molecules, which is called drug/protein promiscuity (Soremekun *et al.* 2019, Gupta *et al.* 2020).

To address the limitations, we propose BindingSiteDTI that performs a differential-scale extraction strategy across the drug and target, which accommodates the scale differences between macro-molecules and micro-molecules. This strategy enables BindingSiteDTI to model the interaction at the substructural level to derive sophisticated interaction features for accurate prediction. Experimental results prove that BindingSiteDTI achieves significant generalizability in DTI prediction.

## 2 Materials and methods

### 2.1 Problem definition

The DTI prediction problem is formulated as follows: Given a set of drugs $D=\{d_{1},d_{2},\ldots ,d_{n}\}$ and a set of target proteins $P=\{p_{1},p_{2},\ldots ,p_{m}\}$, each DTI instance is represented by a tuple $\left(d_{i},p_{j},y_{ij}\right)$, with $y_{ij}\in \{0,1\}$ denoting the binary interaction label for drug $d_{i}$ and protein $p_{j}$. Our approach constructs drug graphs $G_{d}$ from SMILES representation and protein graphs $G_{p}$ from PDB files. The objective is to learn a method $M(G_{d},G_{p})\to \{0,1\}$ that accurately predicts the interaction label from the graph representations. The evaluation on the test set is conducted on unseen instances, comprising drug-protein pairs not previously encountered.

### 2.2 BindingSiteDTI framework

**Figure 2.**
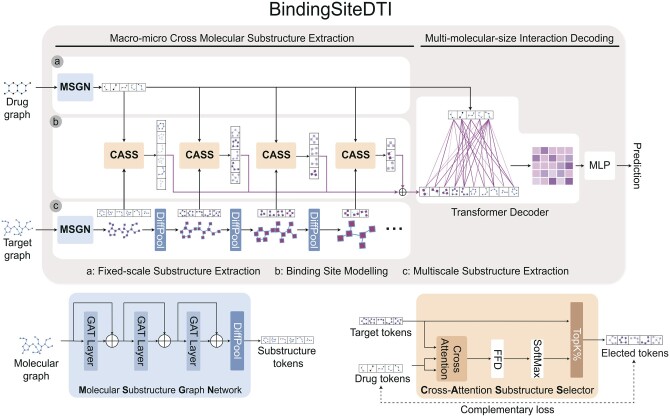
BindingSiteDTI includes two main modules: MMCMSE and MMSID. MMCMSE extracts fixed-scale substructures (block a) for the drug and multiscale substructure for the target (block c). Top K% relevant substructure between drugs and targets will be selected as the preliminary step of interaction modelling (block b). MMSID will learn the hidden relationship between different drugs and target substructures as the interaction feature for downstream prediction.

The overview of BindingSiteDTI is illustrated in Fig. 2, BindingSiteDTI is an end to end DTI classification network where input is the constructed drug graph and target graph, and output is the predicted interaction. There are two major modules, Macro-micro Cross Molecular Substructure Extraction(MMCMSE) extracts a fixed-scale substructural tokens of drug and substructural tokens of target in multiple scales, and initially captures interactions by selecting important tokens based on the measurement of similarity. Multi-molecular-size Interaction Decoding (MMSID) module builds upon the initial interactions to further extract high-level interaction features, thereby unveiling hidden substructure-wise relationships that contribute to improved generalization.

#### 2.2.1 Graph construction

**Drug graph construction:** The drug graph $G_{d}=(V_{d},E_{d})$ is constructed from the SMILES representation, with $V_{d}$ signifying the atoms of the molecule and $E_{d}$ the chemical bonds between them. Each node $v_{di}\in V_{d}$, is encoded with attributes such as element type, chirality, bond connectivity (degree), formal charge, hydrogen attachment, radical electrons, hybridization, aromaticity, and ring membership. For each edge $e_{dj}\in E_{d}$, is represented by a feature vector $fe_{dj}$, delineating bond type, direction, stereochemistry, and conjugation status (Zhu *et al.* 2022).

**Protein graph construction:** For constructing the protein graph $G_{p}=(V_{p},E_{p})$, we utilize the PDB file, with $V_{p}$ as the embedded protein residue types and $E_{p}$ is the connection between these residues. During the preprocessing, protein pockets will be set as the sub-labels of each node.

Detailed statistics and feature definition of constructed graph data are provided in Supplementary Materials.

#### 2.2.2 Macro-micro cross molecular substructure extraction

Algorithm 1 Cross Attention Substructure Selector (CASS)

 *CA*: Cross Attention

 *FFD*: Feed Forward Network

 *k*: Proportion of the Top K%

 **Input**: $T_{M}$: Main Graph Tokens

     $T_{R}$: Reference Graph Tokens

 **Output**: $T_{p}^{\prime }$: Subset of Main Graph Tokens

1: $W⇐\mathrm{CA}(T_{M},T_{R})$

2: $M^{\prime }⇐T_{M}⊗W$

3: $S⇐\mathrm{FFD}(M^{\prime })$  # Learnable selection network.

4: $S^{\prime }⇐\text{Softmax}(S)$

5: $T_{p}^{\prime }⇐\text{TopK}\%(T_{M},S^{\prime },k)$  # Subset of original $T_{M}$.

MMCMSE is the core module which performs binding site modelling in differential-scale, extracting the most correlated substructural tokens of two input graphs. A fixed-scale substructure will be extracted for drug while multiscale of substructure for protein. This method allows network to access different pairs of drug and target input without the negative influence caused by extremely differential molecular size, and also maintain the scale difference between micro-molecule and macro-molecule. Different with existing two-stage DTI network, during the MMCMSE, BindingSiteDTI preliminarily model the interaction based on a set of Cross-Attention Substructure Selector (CASS) modules to identify and select important substructures based on the similarity between tokens.

**Molecular substructure graph network:** MSGN is a graph neural network that accepts an input graph and generates substructure tokens. The feature extraction process can be described by the following steps: For a given graph representation of a molecular graph G, the node feature vector f is provided. To learn the chemical structure of the input molecule, a set of graph attention layers are adopted to update the node feature.

The feature vector after the *i*th GAT layer is defined as:

(1) $$f^{\left(i+1\right)}={\text{GAT}}^{i}(G,f^{\left(i\right)})$$

The layer number of GAT is a hyper-parameter which is fixed during the training and testing stage. To avoid the over-smoothing problem occurred when the inputted graph is a small molecule. The residual connection is adopted:

(2) $$f^{\left(i+1\right)}=f^{\left(i+1\right)}+f^{\left(i\right)}$$

Finally, to construct substructure tokens, we adopt DiffPool (Ying *et al.* 2018) on the updated node feature $f^{n}$, which gives:

(3) $$T=\text{DiffPool}(G,f^{n})$$

**Cross-attention substructure selector:** CASS takes two graph data inputs: a main graph tokens $T_{M}$ and a reference graph tokens $T_{R}$. In BindingSiteDTI, the protein graph is the main graph with the corresponding token $T_{p}$, while the drug graph is the reference graph with tokens $T_{d}$. The function of CASS is to identify the most crucial main graph tokens referring to the reference graph tokens, the detailed processes are provided in Algorithm 1.

(4) $$T_{p}^{\prime }=\text{CASS}(T_{p},T_{d},k)$$

Here, $T_{p}^{\prime }$ denotes the subset of protein tokens from the protein graph after selection, which are the top k% of these tokens according to the *S* to the drug molecule:

(5) $$S=\text{Softmax}(\text{FFD}(\text{CA}(T_{p},T_{d})))$$

*S* is the cross attention scores between each substructure token of protein $T_{p}$ and drug $T_{d}$, a feed-forward network FFD to learn the deeper relationship between each substructure. Softmax is performed to normalize the scores. These scores are crucial for the model to prioritize the substructures that are most likely to interact with the drug.

After obtaining *S*, CASS selects the top k% tokens based on *S*, to elect important substructures:

(6) $$T_{p}^{\prime }=\text{TopK}\%(T_{p},k,S)$$

The elected tokens are then aggregated into a feature vector for similarity constraint. for aggregation, scatter add are adopted as follow:

(7) $$o_{i}=o_{i}+\sum_{j:S_{j}=i}s_{j}$$

In this equation, $o_{i}$ represents the graph feature vector which aggregate nodes together by summing the node feature $s_{j}$. In our case, $s_{j}$ are the elected tokens $T_{p}^{\prime }$, and $o_{i}$ is the *i*th stage protein representation vector $v_{p}{\;}_{i}$, which utilized for compute complementary loss.

**Complementary loss:** When a drug binds to a protein, substructural similarities can enhance the interaction. On the contrary, if there is no interaction, similar substructures may still be present; however, binding may not occur due to other factors, such as incompatible chemical properties.

The complementary loss, denoted as *CL*, is formulated to enforce a similarity constraint during instances of active interaction while preventing the enforcement of divergence on inactive DTI samples. The loss is formally defined as follows:

(8) $$CL=\sum_{i}y_{i}\cdot ||v_{d}{\;}_{i}-v_{p}{\;}_{i}|{|}^{2}$$

where *i* indicates the index traversing all corresponding elements in the label $y_{i}$ and output tensors. $||v_{d}{\;}_{i}-v_{p}{\;}_{i}|{|}^{2}$ is the squared Euclidean distance that indicates the disparity between the drug’s and the protein’s representation vectors.

For the purpose of similarity assessment, the global representation vector v is computed by applying sum pooling across all the nodes in the input graph that have been selected based on the top k% scores:

(9) $$v=\sum_{j:S_{j}=i}T_{j}$$

The total training loss *L* is computed by combining the Binary Cross-Entropy *B* loss with *CL*, weighted by a coefficient *w*. This formulation is expressed as:

(10) L=B+w·CL

In our method, the weight *w* assigned to the *CL* is a hyper-parameter. We recommend setting it to 0.01 to strike a balance between the importance of classification accuracy and the enforcement of similarity constraints, thereby optimizing overall performance.

**Drug substructure extraction:** Drug graph $G_{d}$ is processed directly by the Molecular Substructure Generation Network (MSGN):

(11) $$G_{d}^{\prime },T_{D}=\text{MSGN}(G_{d},f_{d})$$

The output of MSGN is the updated drug graph $G_{D}^{\prime }$ and extracted substructure tokens $T_{D}$, this step will provide a fixed scale of substructure extraction for drug molecules.

**Protein substructure extraction and binding site modelling:** Protein graph $G_{p}$ will have a more complicated process to achieve the extraction of multiscale substructure tokens. At the beginning, we perform message passing on the input graph to enrich the hidden feature of each node:

(12) $$G_{p}^{\prime },T_{p}=\text{MSGN}(G_{p},f_{p})$$

Afterward, multiple DiffPool layers are performed to extract multiscale substructures:

(13) $$G_{p}^{i+1},T_{p}^{i+1}={\text{DiffPool}}^{i}(G_{p}^{i},T_{p}^{i})$$

A set of CASS modules will be performed between $T^{i}{\;}_{p}$ and $T_{D}$ to elect the most relevant top k% substructure tokens, enhancing the generalizability of the network:

(14) $$T^{i}{\;}_{p}^{\prime }={\text{CASS}}^{i}(T^{i}{\;}_{p},T_{D},k)$$

Ultimately, we obtain the protein’s final representation by concatenating all intermediate substructure tokens:

(15) $$T_{P}=\text{concat}(T_{p}^{1}{\;}^{\prime },T_{p}^{2}{\;}^{\prime },\ldots ,T_{p}^{n}{\;}^{\prime })$$

**Figure 3.**
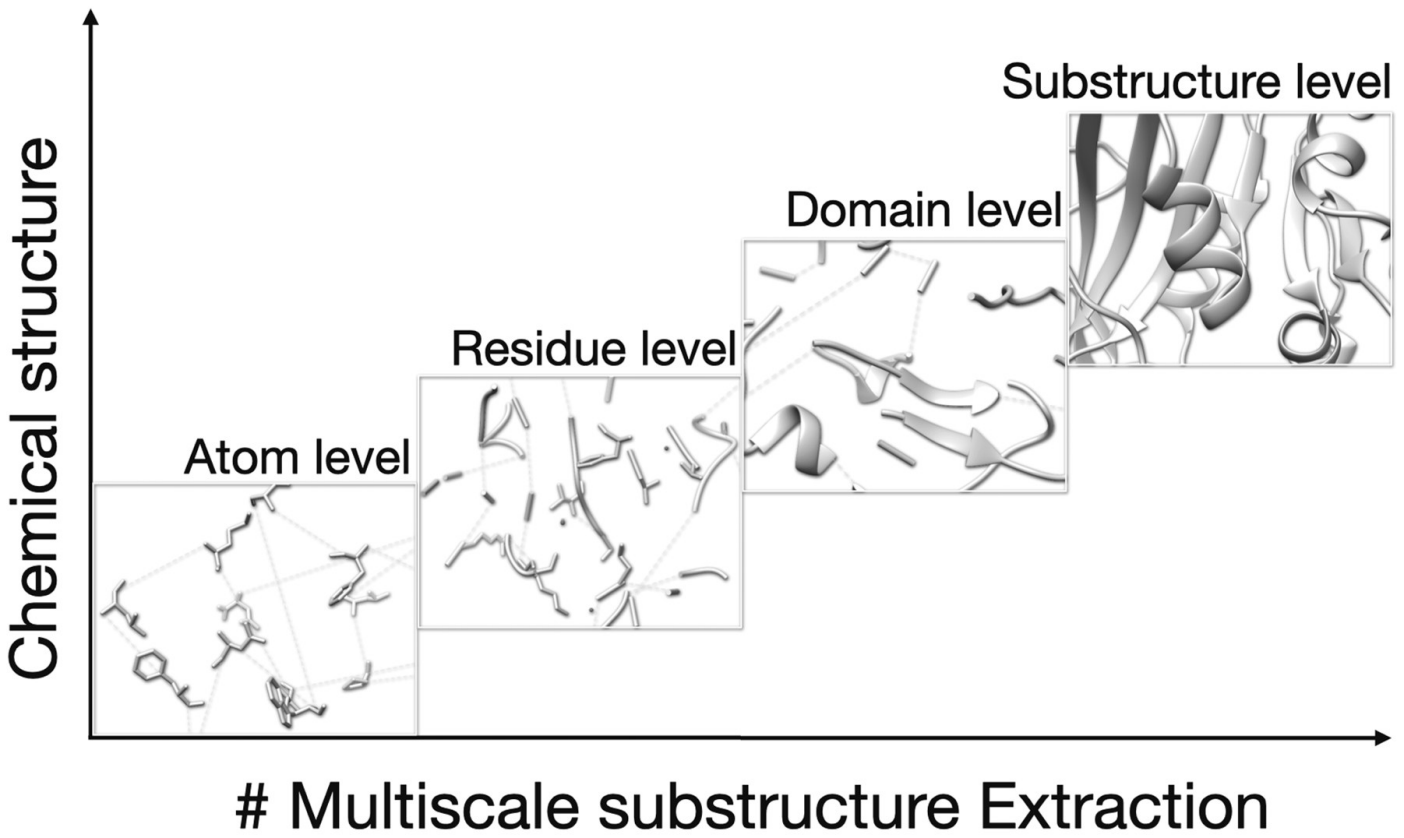
Protein tokens visualization: we trace protein tokens back to original nodes and visualized by UCSF Chimera (Pettersen *et al.* 2004)

To verify the effectiveness of this module, we backtrack different scale of protein tokens to original nodes, and visualized the substructure in Fig. 3. At an early stage, tokens shown by Chimera are just at the atom level. With the increasing number of pooling processes, protein tokens will represent more hyper structures like residues, domains, motifs, substructures etc.

#### 2.2.3 Multi-molecular-size interaction decoding

After BindingSiteDTI extracted the substructure tokens of the drug and target, MMSID will model the interaction features F, which is defined as the hidden relationships of the substructures between the drug and the protein on multiple scales.

The input of MMSID are fixed-scale tokens $T_{D}=\{T_{d}{\;}_{1},T_{d}{\;}_{2},\ldots ,T_{d}{\;}_{i}\}$ and multiscale protein substructure tokens $T_{P}=\{T_{p}^{1}{\;}^{\prime },T_{p}^{2}{\;}^{\prime },\ldots ,T_{p}^{j}{\;}^{\prime }\}$.

The relationship between drug tokens $T_{D}$ and protein tokens $T_{P}$ is defined as the high-level interaction feature F, to model the interaction, we adopt the Transformer Decoder:

(16) $$F=\text{TransformerDecoder}(T_{D},T_{P})$$

Subsequently, the interaction scores are subjected to normalization via softmax to produce the weights w:

(17) $$w_{i}=\frac{e^{F_{i}}}{\sum_{j=1}^{K}e^{F_{j}}}$$

These weights are subsequently utilized to calculate the weighted interaction features:

(18) $$F^{\prime }=w⊙F$$

Here, ⊙ represents element-wise multiplication. $F^{\prime }$ is a matrix encapsulates the weighted interaction scores. To facilitate the inclusion of all the interaction features between various substructures, we flatten $F^{\prime }$ into a vector f:

(19) $$f=\text{Flatten}(F^{\prime })$$

Lastly, the flattened feature vector f is then passed through a multilayer perception (MLP) for prediction:

(20) $$\hat{y}=\text{MLP}(f)$$

### 2.3 Implementation

BindingSiteDTI is developed using Python 3.9.15, PyTorch 2.0.1, and the TorchDrug 0.2.0. The model is executed on an NVIDIA A100 80GB PCIe GPU, under Linux-5.4.17. Our default configuration includes three GAT layers in MSGNs with an 80D representation. The number of drug tokens is set to 8, the number of target tokens are extracted at [1024, 512, 256, 128] during MMCMSE. By default, we recommand to select top 30% of tokens will be selected at each stage,where the experiment and discussion are provided in Supplementary Materials. For MMSID, the architecture comprises 3 layers with an 8-head decoder and a 256D hidden layer. MLP with one hidden layer is employed as the classifier. Training is performed using Adam optimizer, with an exponential learning rate scheduler, having an initial learning rate of 0.001 and decrease 10% during each epoch. Batch size is set as 128. Early stopping is adopted.

## 3 Result

### 3.1 Evaluation strategies and settings

To comprehensively emulate various scenarios, we use three latest symbolic settings in different popular datasets, including human (Liu *et al.* 2015), BindingDB (Gilson *et al.* 2016), and DUD-E (Mysinger *et al.* 2012), the statistics of the datasets are illustrated in Table 1.

**Table 1.** Statistics of datasets.

Datasets	Drugs	Targets	D–T pairs	Active	Inactive
DUD-E	22 886	102	1 429 790	22 645	1 407 145
Human	2726	2001	6728	3364	3364
BindingDB	989	383	8536	39 747	31 218

**BindingDB dataset** (Gao *et al.* 2018): We follow the latest BindingDB setting (Gao *et al.* 2018, Zheng *et al.* 2020, Yazdani-Jahromi *et al.* 2022). To address the imbalance issues, in addition to AUROC, we also report the AUPRC, the F1 score and the Matthews correlation coefficient (MCC).

**Human dataset** (Liu *et al.* 2015): To assess the model’s capacity for interaction prediction while mitigating representation overfitting, we follow Bai *et al.* (2023) to use the same dataset splits and experimented with five random seeds, reporting the average values of AUROC and AUPRC.

**DUD-E dataset** (Mysinger *et al.* 2012): To evaluate the model generalizability for predicting DTI across different protein families, we adhere to the widely accepted setting in DUD-E (Zheng *et al.* 2020, Yazdani-Jahromi *et al.* 2022). Threefold cross-validation is performed to ensure that active drugs of the same target are allocated to the same data fold. We report the average ROC enrichment metrics (Ragoza *et al.* 2017) with varying coefficients to facilitate meaningful comparisons.

### 3.2 Performance comparison on BindingDB dataset

According to the results listed in Table 2, BindingSiteDTI outperforms existing methods in both scenarios of predicting seen and unseen targets’ interactions, in terms of AUROC, AUPRC, MCC, and F1 score.

**Table 2.** BindingDB dataset comparison.

Method	AUROC	AUPRC	MCC	F1
Seen targets				
DeepDTA	0.926	0.916	0.682	0.847
DeepConv-DTI	0.982	0.979	0.866	0.934
TransformerCPI	0.636	0.871	0.581	0.797
GraphDTA	0.932	0.926	0.717	0.863
MolTrans	0.919	0.903	0.722	0.865
BridgeDPI	0.960	–	–	–
AttentionSiteDTI	0.970	–	–	–
DrugBAN	**0.988**	0.984	0.902	0.951
PerceiverCPI	0.983	0.979	0.893	0.947
**BindingSiteDTI (ours)**	**0.988**	**0.986**	**0.904**	**0.952**
Unseen targets				
DeepDTA	0.770	0.839	0.438	0.831
DeepConv-DTI	0.851	0.914	0.543	0.833
TransformerCPI	0.636	0.748	0.054	0.068
GraphDTA	0.748	0.825	0.356	0.809
MolTrans	0.818	0.850	0.550	0.848
BridgeDPI	0.820	–	–	–
AttentionSiteDTI	0.940	–	–	–
PerceiverCPI	0.894	0.927	0.565	0.810
DrugBAN	0.877	0.921	0.610	0.815
DrugBAN with DANN	0.924	0.951	0.685	0.851
DrugBAN with CDAN	0.950	0.973	0.747	0.881
**BindingSiteDTI (ours)**	**0.964**	**0.980**	**0.805**	**0.929**

The best model is shown in bold and the second best model is underlined.

When dealing with seen target interactions, BindingSiteDTI exhibits superior performance across all utilized metrics, demonstrating the best discriminability.

For unseen targets, BindingSiteDTI consistently surpasses all competing approaches in every metric. Furthermore, BindingSiteDTI achieves the highest AUPRC (0.980, compared to 0.973 for DrugBAN with CDAN), the highest MCC (0.805, compared to 0.747 for DrugBAN with CDAN), and the highest F1 score (0.929, compared to 0.881 for DrugBAN with CDAN). Compared to the enhanced versions of DrugBAN that integrate CDAN or DANN for domain adaptation, BindingSiteDTI demonstrates remarkable generalization capabilities in predicting interactions with unseen targets.

### 3.3 Performance comparison on DUD-E dataset

As shown in Table 3, our model outperformed other models in terms of RE 0.5%, RE 1.0%, RE 2.0%. At RE 0.5%, BindingSiteDTI achieved an average score of 110.55, outperforming the second-best model. Similarly, at 1.0% and 2.0% RE, our model continued to lead, scoring 64.34 and 36.80 respectively.

**Table 3.** DUD-E dataset comparison.

Method	0.5%	1.0%	2.0%	5.0%
NN Score	4.166	2.98	2.46	1.891
RF-score	5.628	4.274	3.499	2.678
Vina	9.139	7.321	5.811	4.444
3D-CNN	42.559	26.655	19.363	10.71
PocketGCN	44.406	29.748	19.408	10.735
DrugVQA	88.17	58.71	35.06	**17.39**
AttentionSiteDTI	101.74	59.92	35.07	16.74
DrugBAN	79.26	49.88	30.25	14.76
DrugBAN with CDAN	80.53	48.98	29.6	14.74
**BindingSiteDTI (ours)**	**110.55**	**64.34**	**36.80**	16.81

The best model is shown in bold and the second best model is underlined.

These results confirm the strong performance of the BindingSiteDTI model in predicting DTI across different protein families. BindingSiteDTI’s superior RE scores across most of the cut-off percentages highlight its proficiency in predicting DTI where the target families are unseen before.

### 3.4 Performance comparison on human dataset

**Figure 4.**
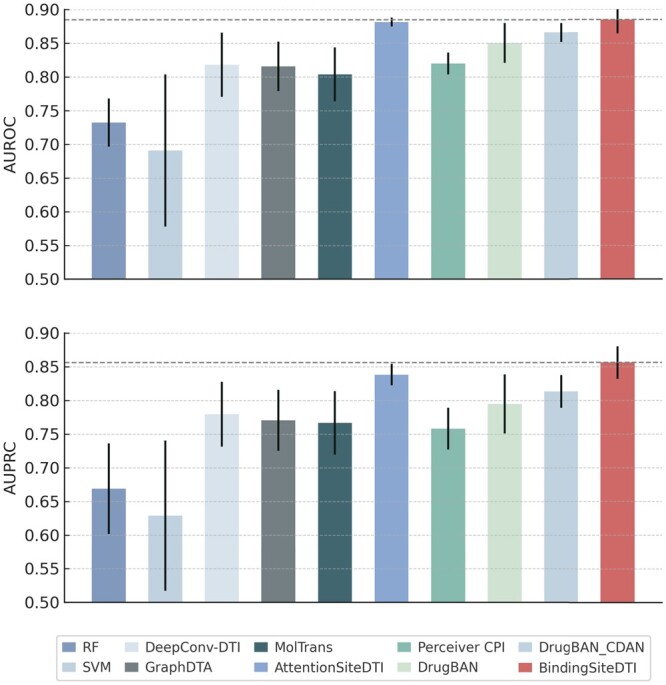
Experiment results on human dataset. Average AUROC and AUPRC are illustrated with error bars of standard deviation.

As illustrated in Fig. 4, our method performed significant results in both AUROC and AUPRC metrics, demonstrates the state-of-the-art performance over the others. Comparing with DrugBAN and PerceiverCPI, our model exhibits better performance. The low standard deviation suggests that the prediction of BindingSiteDTI suggests that it is less sensitive to random factors.

### 3.5 Ablation study

We conducted ablation study to assess the contributions of different components, as illustrated in Table 4. Removing the interaction modelling module resulted in a significant drop in performance, emphasizing its role in capturing essential patterns between drug and target entities. Excluding complementary loss led to decreased performance. The top K% selection demonstrated its importance by prioritizing relevant DTIs. Removal of the MMCMSE module also affected performance. In summary, our ablation study highlights the importance of MMSID, complementary loss, top K% selection, and MMCMSE module in achieving improved performance.

**Table 4.** Ablation study result. The best model is shown in bold.

Model	AUROC	AUPRC
BSDTI without CASS	0.8744	0.8296
BSDTI without comp loss	0.857	0.773
BSDTI with comp loss all time	0.8571	0.774
BSDTI without MMSID	0.4856	0.4487
BSDTI without TopK% selection	0.8098	0.7761
BSDTI without MMCMSE	0.8723	0.8068
**BSDTI (full version)**	**0.8971**	**0.8514**

The best model is shown in bold.

### 3.6 Interpretability of BindingSiteDTI

**Figure 5.**
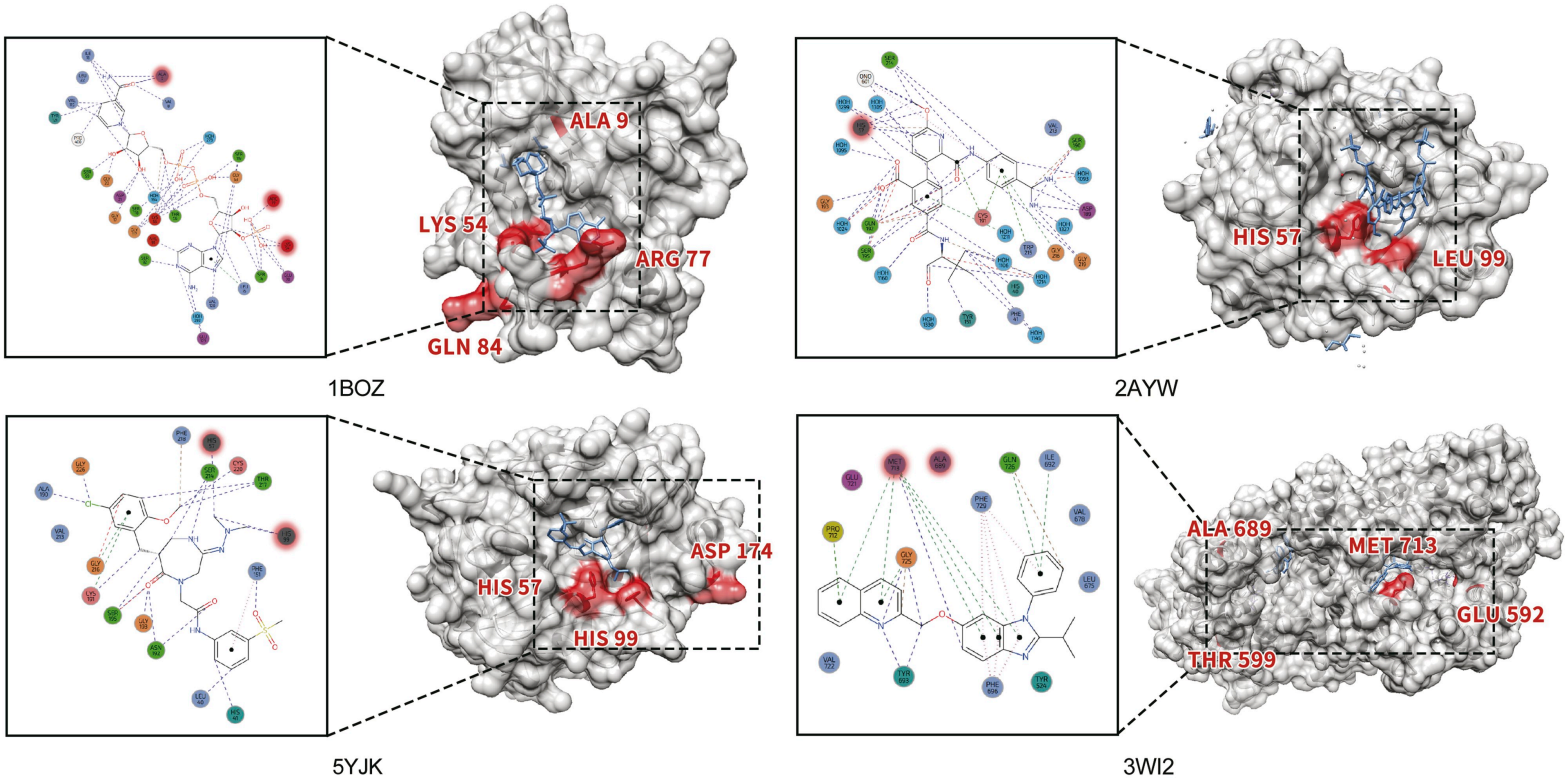
The visualization result of BindingSiteDTI during prediction unseen drug–target pairs, the highlighted red region is top *k* attention weighted substructure of target by the proposed method. For each case, we plot the ground truth of the binding site on the left and our visualization on the right.

In this section, we visualized 4 different DTI samples, target substructures with significant attention weights are highlighted based on our trained model. We use UCSF Chimera (Pettersen *et al.* 2004) to visualize the PDB files and annotate the binding site. The results are shown in Fig. 5.

**PDB ID: 1BOZ:** Our model correctly highlights the protein residues LYS54 and ARG77, where these two regions interact with the O2X, C1B, and O3X of the ligand.

**PDB ID: 2AYW:** BindingSiteDTI highlights HIS57, which interacts with C17 of ONO and contains electrostatic interactions with C58, O44, N16, and O28.

**PDB ID: 5YJK:** Residue HIS57 and HIS99 are correctly highlighted. Both are aromatic and bind to 8VX through electrostatic interactions, connecting with C68 and C63.

**PDB ID: 3WI2:** Visualization result shows BindingSiteDTI focused on MET713, which is hydrophobic and has interaction with substructure of P98. ALA689 is also correctly highlighted, where it hydrophobically binds to the ligand.

These outcomes align with our expectations and are key factors in why our model outperformed many existing methods in this area. Based on MMCMSE module, we were able to uncover significant insights into drug target interactions.

## 4 Conclusion

In this study, we proposed BindingSiteDTI, which incorporates a differential-scale scheme to better model the interaction between drugs and targets. The experimental results support that BindingSiteDTI has strong generalizability and interpretability. Overall, BindingSiteDTI is a powerful approach in the drug discovery process, offering improved predictive accuracy and versatility that may significantly benefit future research and development in the field.

## Supplementary Material

btae308_Supplementary_Data

## Data Availability

All data used in the study are from public resources. Human-cold dataset is available at https://github.com/peizhenbai/DrugBAN BindingDB-IBM is available at https://github.com/IBM/InterpretableDTIP and DUD-E is available at https://dude.docking.org/.
