# Supplementary material for: BindingSiteDTI: differential-scale binding site modelling for drug–target interaction prediction
Source: Bioinformatics. 2024 May 10;40(5):btae308. doi: 10.1093/bioinformatics/btae308 (PMC11256917; doi:10.1093/bioinformatics/btae308)
Supplement: btae308_Supplementary_Data [file btae308_supplementary_data.pdf]

Supplementary Materials for "BindingSiteDTI: Differential-scale Binding Site Modeling for Drug-Target Interaction Prediction"

Feng Pan[1], Chong Yin[1], Si-Qi Liu[1,2], Tao Huang[3], Zhaoxiang Bian[3], and Pong C. Yuen[1,*]

[1]Department of Computer Science, Hong Kong Baptist University, Kowloon, Hong Kong
[2]Shenzhen Research Institute of Big Data, Shenzhen, China
[3]School of Chinese Medicine, Hong Kong Baptist University, Kowloon, Hong Kong
[*]Corresponding author: pcyuen@comp.hkbu.edu.hk

March 2024

1 K-value of Cross-Attention Substructure Selector

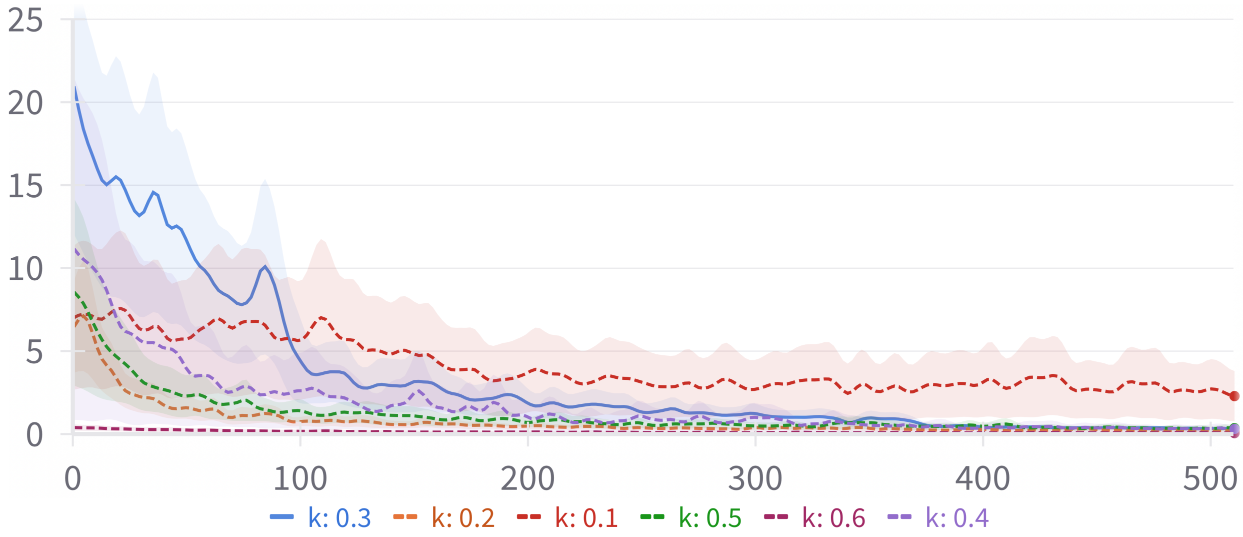

Figure 1: Impact of k-value on binding site modelling.

Cross-Attention Substructure Selector is the core module for BindingSiteDTI, which selects the top k% structural similar tokens between fixed drug tokens and multiscale protein tokens. In this module, k-value is the significant factor directly influence the binding site modelling. Here we perform some experiments and discuss about how to find the reasonable k-value.

Figure 1 illustrated the complementary loss of different BindingSiteDTI settings categorized by a set of reasonable k-value options. The mean values for each category are plotted with standard error. Based on this diagram, when $k = 0.3$, the descending trend of the complementary loss is most pronounced, and the convergence is optimal in the end. This shows that, throughout the process, the model has learned how to select and model binding sites effectively. Therefore, we recommend adopting 30% as the preferred choice for the top k%.

2 Statistics of Constructed Graph Data

We illustrate the statistics for nodes and edges across all datasets in Tables 1, 2, and 3, which provide the details of the pre-processed graph data in BindingDB, human, and DUD-E, respectively. The data reveal extreme variability for graph size. This observation underlines the rationale for our method where differential-scale modelling is necessary for DTI prediction.

	Min	Max	Mean	Median	Std Dev
Drug Nodes	4	72	30.39	30	7.84
Drug Edges	6	156	66.79	68	17.47
Protein Nodes	48	7532	834.17	666	613.55
Protein Edges	7	20505	2802.69	2276	2181.37

Table 1: BindingDB Dataset Statistics

	Min	Max	Mean	Median	Std Dev
Drug Nodes	1	184	19.49	19.0	16.12
Drug Edges	0	384	40.36	38.0	35.51
Protein Nodes	33	9787	1054.14	733.0	971.96
Protein Edges	2	27781	3280.83	2109.0	3222.87

Table 2: Human Dataset Statistics

	Min	Max	Mean	Median	Std Dev
Drug Nodes	2	52	28.53	29.0	6.06
Drug Edges	2	122	61.91	64.0	13.86
Protein Nodes	245	3527	774.68	537.0	604.88
Protein Edges	439	11016	2099.27	1364.0	1863.19

Table 3: DUD-E Dataset Statistics

2.1 Definition of constructed molecular graph

As we construct the graph data for drug and proteins, In this section we provide the detailed information about the definition of node and edges of these graphs. The feature dimension of nodes and edges are mentioned in Table 4.

Drug Graph Definition. The drug node feature comprises a 67-dimensional embedding encoding various chemical properties including atom type, chirality, bond degree, formal charge, hydrogen attachment, radical electrons, hybridization, aromaticity, and ring structure. Conversely, the drug edge feature is a 64-dimensional embedding reflecting bond type, direction, stereochemistry, and conjugation status.

Protein Graph Definition. The protein node feature is represented by a 64-dimensional one-hot embedding of residue types. Edge features are omitted because (1) protein edges merely indicate residue connectivity, and (2) the distances between protein residues are dynamic in living organisms.

	Node Dimension	Edge Dimension
Drug Graph	67	18
Protein Graph	64	NA

Table 4: Node and Edge Dimensions
